# Effectiveness and Safety of Topical Chlorhexidine and Vitamin E TPGS in the Treatment of *Acanthamoeba* Keratitis: A Survey on 29 Cases

**DOI:** 10.3390/jcm9113775

**Published:** 2020-11-23

**Authors:** Ciro Caruso, Daniela Eletto, Michele Rinaldi, Luigi Pacente, Salvatore Troisi, Francesco Semeraro, Roberto dell’Omo, Ciro Costagliola

**Affiliations:** 1 Corneal Transplant Centre, Pellegrini Hospital, 80134 Naples, Italy; cirocarusoeye@gmail.com (C.C.); pacente@oculum.it (L.P.); 2 Department of Pharmacy, University of Salerno, 84084 Fisciano, Italy; 3 Eye Clinic, Multidisciplinary Department of Medical, Surgical and Dental Sciences, University of Campania “Luigi Vanvitelli”, 80131 Naples, Italy; michrinaldi@libero.it; 4 Department of Ophthalmology, Salerno University Hospital, 84131 Salerno, Italy; salvatore.troisi@gmail.com; 5 Department of Medical and Surgical Specialties, Radiological Sciences and Public Health, University of Brescia, 25123 Brescia, Italy; francesco.semeraro@unibs.it; 6 Department of Medicine and Health Science, “V. Tiberio”, University of Molise, 86100 Campobasso, Italy; roberto.dellomo@unimol.it (R.d.); ciro.costagliola@unimol.it (C.C.)

**Keywords:** *Acanthamoeba* keratitis, chlorhexidine, vitamin E TPGS, ocular infections, drug delivery systems

## Abstract

This study aimed to test the effectiveness of a solution of chlorhexidine (CHX) and D-α-tocopheryl polyethylene glycol succinate (Vitamin E TPGS or TPGS) in the treatment of *Acanthamoeba* keratitis (AK) via a prospective, interventional case series study. Twenty-nine consecutive patients with AK were enrolled. At baseline, best-corrected visual acuity (BCVA), slit lamp examination, confocal microscopy, and polymerase chain reaction (PCR) were performed. Topical therapy with CHX 0.02% and VE-TPGS 0.2% was administered hourly/24 h for the first day, hourly in the daytime for the next three days, and finally, every two hours in the daytime up to one month. BCVA and ocular inflammation were recorded after two weeks, four weeks, and three months from baseline. Mean logMAR BCVA significantly improved at two weeks (0.78) compared to baseline (1.76), remaining stable over time (0.80 at four weeks, 0.77 at three months). Ocular inflammation improved in 14 eyes at 2 weeks, with further slow improvements in all cases. At three months, no patient had signs of corneal inflammation. The presence of corneal scars was first recorded at the two-week follow-up, with an enlargement at the four-week follow-up. At the three-month follow-up, 19 eyes still showed corneal opacities. In conclusion, the tested solution was shown to be effective for the treatment of AK. Furthermore, it might represent a good first-line treatment.

## 1. Introduction

*Acanthamoeba* spp. are amphizoic and ubiquitous protist, found in many sources like fresh water and seawater, soil, dust, tap water, air-conditioning units and sewage systems [1]. Such a variety in sources is likely due to differences among countries in the types of contact lens, in the contamination of domestic and swimming pool water, and in the use of diagnostic tests for *Acanthamoeba* keratitis (AK). The free-living amoebas belonging to family *Acanthamoeba* comprises more than 24 species, classified in 20 different genotypes (T1–T20) according to the different sequences in the 18S rRNA gene [2]. Most of the AK cases have been related to genotype T4 which is therefore considered the most predominant genotype.

The applied therapy is often ineffective due to diagnostic mistakes, various pathogenicity of Acanthamoeba strains and high resistance of cysts to drugs. As opportunistic pathogens, *Acanthamoeba* spp. cause infections in several organs in humans, like lungs, liver, kidneys, sinuses, spleen, heart, adrenal glands, skin, and eye. Despite the advances over the years, diagnosis of *Acanthamoeba* spp.-induced infections quite challenging and the efficacy of the current treatments is often poor [2,3]. So far, the main diagnostic methods consist in the analysis of corneal scraping by confocal microscopy, culturing into a dish of *Escherichia coli* plated over non-nutrient agar, and polymerase chain reaction (PCR) [2].

Among the infections, AK is a serious vision-threatening corneal infection, first recognized in 1974 [4]. Users of contact lenses are at the highest risk of infection, and the incidence of the disease ranges from one to two cases per million people in the United States to 1 per 30,000 contact lens users in the United Kingdom [5,6]. This wide variation in incidence is probably due to country differences in the types of contact lens, contamination of domestic and swimming pool water, and in the use of diagnostic tests for AK. However, the increasing use of contact lenses justifies the growth of incidence/year of AK over time. A compromised immune system also increases the risk, whereas no sex predilection has been reported [7].

AK is a clinical entity that is difficult to diagnose and treat. The clinician should have a high suspicion of AK when keratitis occurs in contact lens users. Typical clinical features are severe pain (inconsistent with clinical signs), associated with light hypersensitivity, blurry vision, and tearing. Moreover, the disease is not responsive to standard first-line treatments. The standard treatments can consist of medical approach which enroll the use of biguanides, diamidines, and corticosteroids, or and surgical approach which includes epithelial debridement, amniotic membrane transplant, and penetrating keratoplasty. However, there are no available therapeutic strategies sufficiently efficient to eradicate both the cystic and trophozoite forms of *Acanthamoeba*; the lack of an effective medical management is associated with a poor prognosis [8]. Some authors suggest that combination therapy appears to be more effective [9,10], although such a choice implies increased costs and the occurrence of severe side effects, such as epithelial toxicity [11,12].

Chlorhexidine (CHX) is a biguanide compound used as an antiseptic agent with topical antibacterial activity. In vitro, CHX shows excellent efficacy against trophozoites and cysts, and has already been used for the treatment of AK, but with a relatively high failure rate [13]. Recently, it has been shown that biguanides do not represent an efficient treatment for AK [14]. This failure could depend on the low drug bioavailability in the deep corneal stroma due to its inadequate penetration.

D-alpha-tocopherol poly (ethylene glycol) 1000 succinate (VE-TPGS) is a water-soluble nonionic surfactant used as a pharmaceutical excipient for drug delivery formulations. It has been demonstrated that VE-TPGS promotes drug penetration into the corneal stroma [15,16], and is a good candidate for the enhancement of CHX stromal availability [17].

This study aimed to test the effectiveness of a solution of CHX and VE-TPGS as monotherapy for the treatment of AK.

## 2. Experimental Section

### 2.1. Materials

A solution based on VE-TPGS (0.2%) and CHX digluconate solution (0.02%) from Iromed Group Rome Italy was tested.

### 2.2. Patients

A prospective, interventional case series study was performed. The setting was the Ophthalmology Department of the Pellegrini Hospital, Naples. Patients enrolled in this study developed AK from April 2018 to April 2019. The study was conducted in accordance with the Declaration of Helsinki and its 1983 revision. Institutional Review Board/Ethics Committee, and Animal Care and Use committee approvals were obtained January 2017 (authorization number 1269). Institutional review board approval was obtained, and a written informed consent was provided to each patient. Any information about demographic characteristics and past medical history including age, gender, and previous treatments was recorded in a detailed questionnaire. All the patients enrolled in this study had an onset of symptoms between one and two weeks before the first examination (time 0). Best-corrected visual acuity (BCVA) and slit lamp examination were recorded. Two different physicians (CCa and LP) examined each patient to obtain an impartial judgement of the clinical condition. AK diagnosis was confirmed by microscopy (Giemsa), PCR, and confocal microscopy (Confoscan 3.4, Nidek Co. Ltd., Gamagori, Japan). Patients with inadequate compliance during the treatment were excluded from the clinical trial.

The 18S rRNA (Rns) gene-based PCR method was enrolled in this study in order to diagnose AK and the primers used were the following: The forward primer 5′-GGCCCAGATCGTTTACCGTGAA-3′, the reverse primer 5′-TCTCACAAGCTGCTAGGGGAGTCA-3′. The PCR program was: 2 min at 95 °C, followed by 35 cycles at 95 °C for 1 min, 60 °C for 1 min, and 72 °C for 1 min and a final extension step at 72 °C for 7 min. The PCR products were analyzed by 1.5% agarose gel and revealed a 460-bp amplicon. The amplicons were purified by commercial kits and sequenced for both strands by the original primers. The sequences obtained were then identified by BLASTn search and compared with EMBL database reference sequences. Finally, each sequence was aligned to the reference sequence by the CLUSTALW 1.8 software in order to confirm the strain. All Acanthamoeba strains identified by PCR belonged to *Acanthamoeba castellanii* (T4), and only a few of them to *Acanthamoeba jacobsi* (T15).

In the present study twenty-nine patients were enrolled. The mean age was 27.03 ± 10.61 years (20 females (68.96%) and 9 males (31.04%)). Sixteen subjects (55.17%) were contact lens users (many of them were used to cleaning or storing contact lens with tap water), seven (24.13%) had a history of ocular trauma with organic material (a plant), and two (6.89%) had a history of contamination with dust or dirty water. Seventeen patients (58.62%) had already undergone a therapy (corticosteroid, anti-bacterial, and anti-inflammatory).

At the first eye check all patients mentioned ocular pain, epitheliopathy, conjunctival vessels dilatation, and stromal infiltrates; five (17.24%) had sub-epithelial infiltrates; nine (31.03%) had satellite stromal infiltrates; eight (27.58%) had ring stromal infiltrates; sixteen (55.17%) had a “round dot in a ring” stromal pattern; 11 had uveitis (37.93%); and three had hypopyon (10.34%). The mean BCVA at the recruitment time was 1.76 logMAR (standard deviation (SD): ±0.47). All the demographic and clinical data are reported in Table 1. As the patients were classified according to the inflammation status, the lesions classified as “ring stromal infiltrates” were those in which the risk for ulceration was highest. This datum (8 cases) is reported in Table 1.

### 2.3. Treatment Protocol

Once diagnosed with AK, all patients received topical treatment with a solution of CHX 0.02% and VE-TPGS 0.2% (Iromed Group, Rome, Italy). The solution was instilled in the affected eye hourly/24 h for the first day; then, every hour in the daytime for the next three days, and finally every 2 h in the daytime for a month. During the first two weeks atropine 1% twice a day was added, whereas during the following two weeks dexamethasone phosphate every 6 h was administered. This treatment was unsuccessful in 7 patients (24.13%), in which the infection worsened; in these patients, other anti-Acanthamoeba compounds (polyhexamethylene biguanide and propamidine), together with anti-bacterial therapy, were prescribed to obtain a more favorable clinical response. These alternative treatments were stopped when no signs of active corneal disease were recorded.

### 2.4. Outcomes

Outcomes were collected at two weeks, four weeks, and three months from baseline. They included BCVA, and the following clinical signs: Clear cornea, corneal scar, epitheliopathy, conjunctival vessels vasodilation, sub-epithelial infiltrates, stromal infiltrates, satellite stromal infiltrates, ring stromal infiltrates, “round dot in a ring” stromal pattern, uveitis, and hypopyon. To simplify the description of the clinical signs, a classification system was adopted. 

Visual acuity (VA): Patients were divided into two groups: (1) BCVA logMAR between 2.07 and 0.30 and (2) BCVA logMAR between 0.29 and 0. Inflammation: Patients were divided into four groups: Stage 0, no ocular inflammation; stage 1, epitheliopathy; stage 2, anterior stromal infiltration; and stage 3, ring infiltration, or deep stromal infiltration. Corneal scar occurrence: Patients were divided into five groups, 0 absence of scar; 1, 2, 3, or 4, presence of corneal scars in one, two, three, or four quadrants, respectively [18].

### 2.5. Statistical Analysis

The results are presented as mean ± SD for quantitative variables and percentage values (%) for categorical variables. VA was reported in logMAR. *p*-values ≤ 0.001 were considered statistically significant.

All statistical analyses were performed using SPSS version 13 (SPSS Inc., Chicago, IL, USA) for Microsoft Windows 7.

## 3. Results

### 3.1. Visual Performance

**Figure 1:**
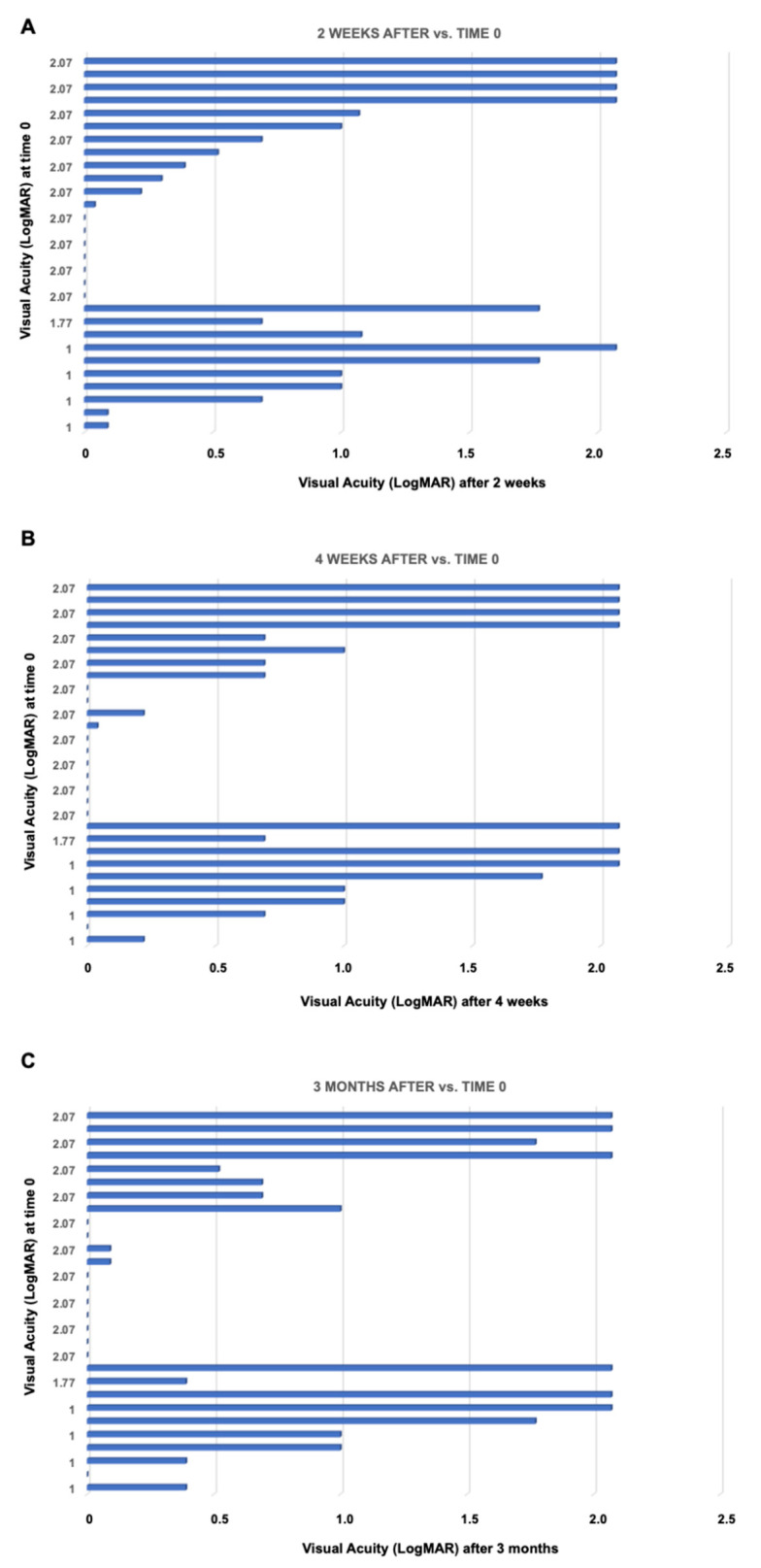
Graphics about overtime changes in visual acuity (expressed in LogMAR) at 2 weeks (**A**), 4 weeks **(B**) and 3 months (**C**) post-treatment.

**Figure 2:**
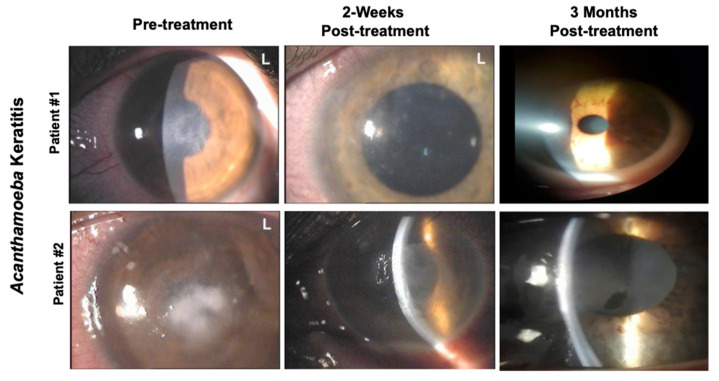
Representative pictures of two patients affected by *Acanthamoeba* Keratitis. Pictures were taken at different time points pre- and post-treatment with a solution of CHX (chlorhexidine) 0.02% and VE-T-GS 0.2%. Patient #1: cornea shows epithelial irregularity and punctate epithelial lesions before treatment. Patient #2: cornea shows a gray-white diffuse stromal infiltrate positioning ring0shaped with stromal thinning prior the treatment.

At baseline (time 0), the BCVA ranged between 1 and 2.07 (mean 1.76) logMAR. After two weeks, the mean BCVA significantly improved compared to baseline (0.78 logMAR, *p* < 0.001). This finding remained substantially stable over time (0.80 at four weeks, 0.77 at three months). In detail, 19 patients had an improvement in their VA (11 shifted in the range between 0.29 and 0), 8 were stable, and 2 worsened at 2 weeks. Between 2 and 4 weeks, 4 patients had an improvement in their VA (2 shifted in the range between 0.29 and 0), 20 remained stable, and 5 worsened (one shifted in the logMAR range between 1 and 2.07, 3 moved to light perception). At the 3 months follow-up, 6 patients had an improvement in their VA, 20 remained stable, and 3 worsened with respect to the value recorded at the 4-week follow-up. These findings are reported in Figure 1 and representative pictures of two patients are reported in Figure 2.

### 3.2. Inflammation

At baseline, all patients had inflammation stage 3. Two weeks later, 14 patients had an improvement in their clinical condition (7 moved to stage 2 and 7 to stage 1). After four weeks, further improvement was recorded (2 patients moved to stage 2, and 9 to stage 1). Corneal inflammation disappeared in 11 patients who reached stage 0. At three months, no patient had signs of corneal inflammation.

**Figure 3:**
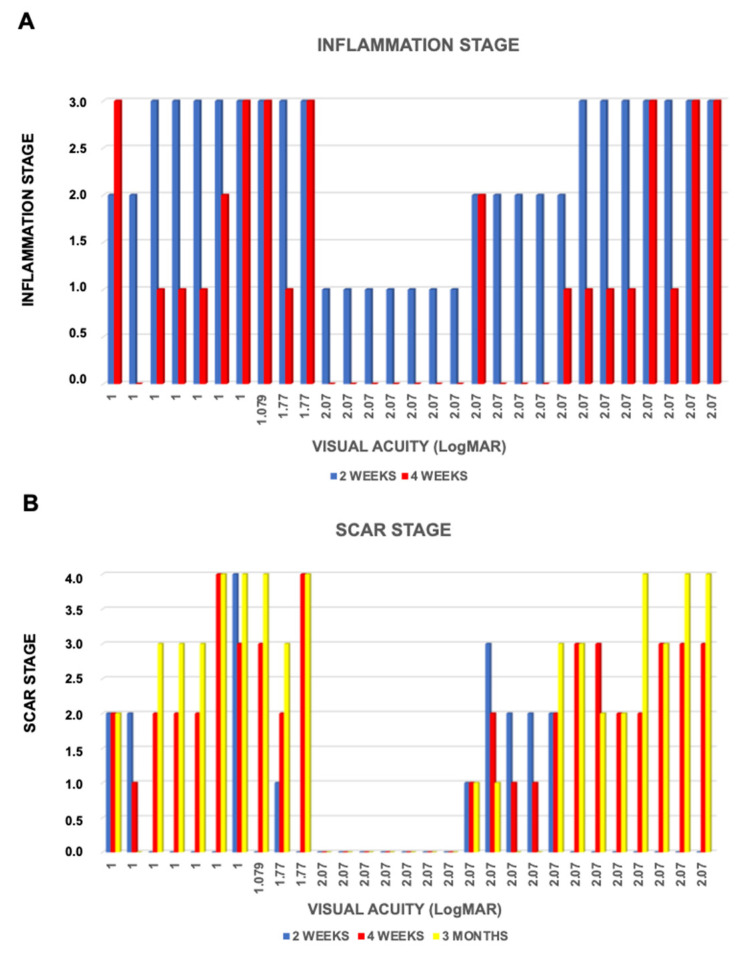
Graphics about overtime changes in inflammation stages (**A**) and scar stages (**B**) at different weeks of treatment.

Corneal epitheliopathy, conjunctival vessel vasodilation, and stromal infiltrates, present in all patients at baseline, greatly improved over time. Sub-epithelial infiltrates showed a peak at two weeks (seven patients), and were still present at four weeks in two of them. All the satellite stromal infiltrates disappeared after four weeks; only three patients still showed ring stromal infiltrates during that follow-up. The “round dot in a ring” stromal pattern largely decreased at four weeks, remaining noticeable only in two patients. Uveitis was found in 11 patients at baseline, and only in 2 patients after 4 weeks. The hypopyon frequency was stable between baseline and four weeks, with a peak at two weeks (five patients). The relationship between VA and inflammation stage at two and four weeks is reported in Figure 3.

### 3.3. Corneal Scarring 

The presence of corneal scars was recorded at the two-week follow-up: Two patients in group 1, five in group 2, one in group 3, and one in group 4. At four weeks, enlargement of the scarring process was seen: 14 patients had a worsening, 9 were stable, and 6 had an improvement; in detail, 7 patients were in stage 0, 4 in stage 1, 9 in stage 2, 7 in stage 3, and 2 in stage 4. Three months later, 5 patients experienced an improvement, 14 were stable, and 10 worsened. There were 10 patients in stage 0, 2 in stage 1, 3 in stage 2, 7 in stage 3, and 7 in stage 4. In the latter group, four patients with a total corneal scarring and light perception underwent surgery (one penetrating keratoplasty and three amniotic membrane appositions). The relationship between VA and scar group at two weeks, four weeks, and three months is reported in Figure 3.

## 4. Discussion

AK is a rare vision-threatening corneal disease that occur mostly in contact lens users, although *Acanthamoeba* spp. can also infect human cornea in non-contact lens users [19]. Thus, in presence of eye pain inconsistent with the degree of keratitis, either in contact lens users or in any case of corneal trauma with consequent exposure to soil or contaminated water, AK should be considered as a potential disease [3]. Other than pain, frequent symptoms are photophobia and tearing and common clinical signs are infiltrate and epithelial defects, anterior stromal ring infiltrates, and radial keratoneuritis [20]. An early AK diagnosis is important, since the earlier the diagnosis, the better the prognosis. When AK is diagnosed too late, the amoebae might penetrate up into the corneal stroma, and a successful therapy could be very difficult [21].

Biguanides are the most effective compounds for the treatment of AK, either alone or in combination, against both cysts and trophozoites [12,22]. In monotherapy, the success rate of biguanides is approximately 80%. However, despite intensive treatment and proven drug susceptibility, there are still many therapeutic failures due to the low drug bioavailability in the deep corneal stroma. In fact, it has been demonstrated that topically administered CHX does not readily penetrate the corneal stroma [23]. To address this issue, novel formulations have been developed using a D-ɑ-tocopheryl polyethylene glycol succinate (TPGS) or VE-TPGS-based strategy [16,24]. This water-soluble molecule is formed by the esterification of vitamin E succinate with polyethylene glycol (PEG) 1000. Its amphipathic properties make this non-ionic surfactant able to form stable micelles in aqueous vehicles and help to solubilize poorly water-soluble molecules. Vitamin E has no irritant effect on the eye, and its antioxidant properties make it beneficial for topical application [25]. Our findings demonstrate that this ophthalmic preparation is very effective, permitting a high accumulation of CHX in human corneas, without the occurrence of toxic keratopathy, a well-known side effect [16,26].

Usually, in our care unit the treatment protocol consists in intensive (day and night) topical application of either a biguanide (polyhexamethylene biguanide-PHMB or chlorhexidine) or a diamidine (propamidine–Brolene; hexamidine), alone or in combination. The treatment is performed for weeks or months, depending on the clinical course.

Topical steroids are given to limit inflammation. Topical antibiotics are used in presence of secondary bacterial super-infection penetrating keratoplasty, but only in presence of corneal irregularity, thinning, and/or scarring severity following complete control of infection. Topical chlorhexidine and vitamin E TPGS in the treatment of AK seems to be more effective and safer as compared to the standard protocol adopted in our clinic.

The clinical outcomes considered were BCVA, inflammation, and corneal scarring. After three months of CHX treatment, 6 patients had an improvement in their VA, 20 remained stable, and 3 worsened. Illingworth et al., in a series of 18 eyes treated with biguanides from the time of diagnosis, reported a VA of 20/30 or better in all patients [27]. More recently, the results from Lim et al. are similar to those obtained in the present case series [12]. A positive correlation between treatment time and BCVA recovery has been reported; in fact, approximately 91% of patients receiving early treatment achieved a BCVA of 6/12 or better [28]. 

The CHX plus VE-TPGS solution resulted to be a good therapeutic approach against Acanthamoeba inflammation. LogMAR BCVA of 0.69 or better was achieved in 18 of 29 eyes (62%), and 0.5 or better in 15 eyes (51.72%). This success rate is comparable to what reported in three other major studies that used combination therapy [11,28,29]. PHMB and propamidine are often used agents. Seal et al. used propamidine for the treatment of AK, with one exception, and showed a recovered 6/9 vision in 11 patients; 1 patient with ring infiltration required a keratoplasty because of a secondary bacterial infection, with graft survival and VA of 6/6 after 2 years [30]. Of the 11 patients reported, only 2 had stromal infiltration; the latter two patients in this series are not comparable to the 29 patients in our study, who presented initial stromal involvement, with or without ring infiltration. We observed a gradual reduction of the inflammation in most of the eyes, corresponding to a progressive scarring of the corneal stroma. Likely, this represents the main reason for the lack of visual improvement (Figure 1 and Figure 3). 

Only four patients (13.79%) required another anti-Acanthamoeba agent. Fifteen patients did not achieve a favorable clinical response (51.72%) after two weeks of treatment; this number decreased to seven eyes (24.13%) after 4 months, and only one had a worsening of keratitis (3.2%). 

The use of corticosteroids in the treatment of AK is still under debate. One study reported that corticosteroids, by inhibiting the encystment of Acanthamoeba trophozoites in vitro, made amoebae more susceptible to anti-Acanthamoeba agents [31]. On the other hand, corticosteroids were shown to increase the rate of treatment failure in AK, while they resulted to produce good outcomes in other papers [32,33,34]. Park et al. reported that the use of corticosteroids delayed the treatment duration compared to a control group and more importantly visual outcomes were not significantly different between the two groups [32]. Taken together, data suggest that a large use of corticosteroids might prolong the whole treatment, but leads to a better visual outcome, probably through the management of inflammatory complications [35].

Chew et al. [36], in a univariate analysis, reported that stromal involvement and initial VA < 20/50 correlated significantly with the need for penetrating keratoplasty and the final VA < 20/25; in multivariate analysis, only initial VA < 20/50 had a significant association. In our study, longer duration of disease before diagnosis, initial VA < 20/40, and stromal involvement were significantly correlated with final VA < 20/25.

Kelley et al. [37] in 6 eyes out of 20 with AK (30%) found secondary glaucoma, which is considered by the authors as the development of elevated intraocular pressure caused by AK, and is correlated to a poor prognosis.

In our study, no patient developed elevated intraocular pressure and it might be related to better inflammation control with the use of corticosteroids.

An important warning coming from our study is the occurrence of AK in cosmetic contact lens users. Fourteen patients used to clean their lenses or contact lens storage box with tap water. Tap water might be a source of *Acanthamoeba*, causing contamination of contact lenses and their storage box [38]. Often, cosmetic contact lenses can spread bacteria or amoebae as they are frequently used improperly. If contact lens were used according to medical guidelines based on disinfection, the incidence of AK due to tap water or improper behavior would be much lower [39].

## 5. Conclusions

In conclusion, a solution based on chlorhexidine and vitamin E TPGS seems to constitute a good candidate for treating *Acanthamoeba* keratitis in a monotherapy protocol or for initial treatment. After an initial response to the aforementioned solution, corticosteroids can be used as adjunctive therapy, depending on the clinical condition. An approach based on combination therapy should be compared to randomized clinical trials in order to establish the treatment efficacy and visual outcomes, on patients affected by different levels of visual acuity and corneal involvement.

Because these results are promising yet preliminary, further research is required to corroborate this encouraging evidence. 

## Figures and Tables

**Table 1:** Demographics and features of the enrolled patients.

	N°	%	Value	Mean ± SD
Total Patients	29	100		
Female	20	68.96		
Male	9	31.04		
Average age (years)			27.03	10.61
Contact lens wear	16	55.17		
Organic material trauma (plant)	7	24.13		
Dust contamination	2	6.89		
Water contamination	1	3.44		
Unknown contamination	3	10.37		
Already in therapy	17	58.62		
Ocular pain	29	100		
Epitheliopathy	29	100		
Peri-keratic vessels dilatation	29	100		
Stromal infiltrates	29	100		
Sub-epithelial infiltrates	5	17.24		
Satellite stromal infiltrates	9	31.03		
Ring stromal infiltrates	8	27.58		
“Round dot in a ring” stromal pattern	16	55.17		
Uveitis	11	37.93		
Ipopion	3	10.34		
Average best corrected visual acuity (LogMAR)			1.76	0.47

% = percentage respect to total; SD = Standard deviation.

## Data Availability

The data that support the findings of this study are available on request from the corresponding author. The data are not publicly available due to privacy or ethical restrictions.

## References

[1] Visvesvara G.S., Moura H., Schuster F.L. (2007). Pathogenic and opportunistic free-living amoebae: Acanthamoeba spp.; Balamuthia mandrillaris, Naegleria fowleri, and Sappinia diploidea. FEMS Immunol. Med. Microbiol..

[2] Lorenzo-Morales J., Khan N.A., Walochnik J. (2015). An update on Acanthamoeba keratitis: Diagnosis, pathogenesis and treatment. Parasite.

[3] Kot K., Łanocha-Arendarczyk N.A., Kosik-Bogacka D.I. (2018). Amoebas from the genera Acanthamoeba and their pathogenic properties. Ann. Parasitol..

[4] Naginton J., Watson P.G., Playfair T.J., McGill J., Jones B.R., Steele A.D. (1974). Amoebic infection of the eye. Lancet.

[5] Stehr-Green J.K., Bailey T.M., Visvesvara G.S. (1989). The epidemiology of Acanthamoeba keratitis in the United States. Am. J. Ophthalmol..

[6] Seal D.V. (2003). Acanthamoeba keratitis update –incidence, molecular epidemiology and new drugs for treatment. Eye.

[7] Bunsuwansakul C., Mahboob T., Hounkong K., Laohaprapanon S., Chitapornpan S., Jawjit S., Yasiri A., Barusrux S., Bunluepuech K., Sawangjaroen N. (2019). Acanthamoeba in Southeast Asia—Overview and Challenges. Korean J. Parasitol..

[8] Maycock N.J., Jayaswal R. (2016). Update on Acanthamoeba Keratitis: Diagnosis, Treatment, and Outcomes. Cornea.

[9] Lam D.S., Lyon D., Poon A.S., Rao S.K., Fan D.S. (2000). Polyhexamethylene biguanide (0.02%) alone is not adequate for treating chronic Acanthameoba keratitis. Eye.

[10] Kitagawa K., Nakamura T., Takahashi N., Oikawa Y., Ikeda T. (2003). A novel combination treatment of chlorhexidine gluconate, natamycin (pimaricin) and debridement for a Acanthamoeba keratitis. Jpn. J. Ophthalmol..

[11] Oldenburg C.E., Acharya N.R., Tu E.Y., Zegans M.E., Mannis M.J., Gaynor B.D., Whitcher J.P., Lietman T.M., Keenan J.D. (2011). Practice patterns and opinions in the treatment of Acanthamoeba keratitis. Cornea.

[12] Bacon A.S., Frazer D.G., Dart J.K., Matheson M., Ficker L.A., Wright P. (1993). A review of 72 consecutive cases of Acanthamoeba keratitis, 1984–1992. Eye.

[13] Lim N., Goh D., Bunce C., Xing W., Fraenkel G., Poole T.R., Ficker L. (2008). Comparison of polyhexamethylene biguanide and chlorhexidine as monotherapy agents in the treatment of Acanthamoeba keratitis. Am. J. Ophthalmol..

[14] Alkharashi M., Lindsley K., Law H.A., Sikder S. (2015). Medical interventions for acanthamoeba keratitis. Cochrane Database Syst. Rev..

[15] Ostacolo C., Caruso C., Tronino D., Troisi S., Laneri S., Pacente L., Del Prete A., Sacchi A. (2013). Enhancement of corneal permeation of riboflavin-5ʹ- phosphate through vitamin E TPGS: A promising approach in corneal trans-epithelial cross linking treatment. Int. J. Pharm..

[16] Paradiso P., Serro A.P., Saramago B., Colaço R., Chauhan A. (2016). Controlled Release of Antibiotics from Vitamin E-Loaded Silicone-Hydrogel Contact Lenses. J. Pharm. Sci..

[17] Caruso C., Porta A., Tosco A., Eletto D., Pacente L., Bartollino S., Costagliola C. (2020). A Novel Vitamin E TPGS-Based Formulation Enhances Chlorhexidine Bioavailability in Corneal Layers. Pharmaceutics.

[18] Ren M., Wu X. (2010). Evaluation of three different methods to establish animal models of Acanthamoeba keratitis. Yonsei Med. J..

[19] Ibrahim Y.W., Boase D.L., Cree I.A. (2007). Factors affecting the epidemiology of Acanthamoeba keratitis. Ophthalmic Epidemiol..

[20] Tananuvat N., Techajongjintana N., Somboon P., Wannasan A. (2019). The First Acanthamoeba keratitis Case of Non-Contact Lens Wearer with HIV Infection in Thailand. Korean J. Parasitol..

[21] Tu E.Y., Joslin C.E., Sugar J., Shoff M.E., Booton G.C. (2008). Prognostic factors affecting visual outcome in Acanthamoeba keratitis. Ophthalmology.

[22] Polat Z.A., Walochnik J., Obwaller A., Vural A., Dursun A., Arici M.K. (2014). Miltefosine and polyhexamethylene biguanide: A new drug combination for the treatment of Acanthamoeba keratitis. Clin. Experimen. Ophthalmol..

[23] Vontobel S.F., Abad-Villar E.M., Kaufmann C., Zinkernagel A.S., Hauser P.C., Thiel M.A. (2015). Corneal Penetration of Poly-hexamethylene Biguanide and Chlorhexidine Digluconate. J. Clin. Exp. Ophthalmol..

[24] Guo Y., Luo J., Tan S., Otieno B.O., Zhang Z. (2013). The applications of Vitamin E TPGS in drug delivery. Eur. J. Pharm. Sci..

[25] Costagliola C., Libondi T., Menzione M., Rinaldi E., Auricchio G. (1985). Vitamin E and red blood cell glutathione. Metabolism.

[26] Carrijo-Carvalho L.C., Sant’ana V.P., Foronda A.S., de Freitas D., de Souza Carvalho F.R. (2017). Therapeutic agents and biocides for ocular infections by free-living amoebae of Acanthamoeba genus. Surv. Ophthalmol..

[27] Illingworth C.D., Cook S.D., Karabatsas C.H., Easty D.L. (1995). Acanthamoeba keratitis: Risk factors and outcome. Br. J. Ophthalmol..

[28] Duguid I.G., Dart J.K., Morlet N., Allan B.D., Matheson M., Ficker L., Tuft S. (1997). Outcome of acanthamoeba keratitis treated with polyhexamethyl biguanide and propamidine. Ophthalmology.

[29] Butler T.K., Males J.J., Robinson L.P., Wechsler A.W., Sutton G.L., Cheng J., Taylor P., McClellan K. (2005). Six-year review of Acanthamoeba keratitis in New South Wales, Australia: 1997–2002. Clin. Exp. Ophthalmol..

[30] Seal D., Hay J., Kirkness C., Morrell A., Booth A., Tullo A., Ridgway A., Armstrong M. (1996). Successful medical therapy of Acanthamoeba keratitis with topical chlorhexidine and propamidine. Eye.

[31] Osato M., Robinson N., Wilhelmus K., Jones D. (1986). Morphogenesis of Acanthamoeba castellanii: Titration of the steroid effect. Investig. Ophthalmol. Vis. Sci..

[32] Park D.H., Palay D.A., Daya S.M., Stulting R.D., Krachmer J.H., Holland E.J. (1997). The role of topical corticosteroids in the management of Acanthamoeba keratitis. Cornea.

[33] O’Day D.M., Head W.S. (2000). Advances in the management of keratomycosis and Acanthamoeba keratitis. Cornea.

[34] Dart J.K., Saw V.P., Kilvington S. (2009). Acanthamoeba keratitis: Diagnosis and treatment update 2009. Am. J. Ophthalmol..

[35] Carnt N., Robaei D., Watson S.L., Minassian D.C., Dart J.K. (2016). The Impact of Topical Corticosteroids Used in Conjunction with Antiamoebic Therapy on the Outcome of Acanthamoeba Keratitis. Ophthalmology.

[36] Chew H.F., Yildiz E.H., Hammersmith K.M., Eagle R.C., Rapuano C.J., Laibson P.R., Ayres B.D., Jin Y.P., Cohen E.J. (2011). Clinical outcomes and prognostic factors associated with Acanthamoeba keratitis. Cornea.

[37] Kelley P.S., Dossey A.P., Patel D., Whitson J.T., Hogan R.N., Cavanagh H.D. (2006). Secondary glaucoma associated with advanced Acanthamoeba keratitis. Eye Contact Lens..

[38] Jeong H.J., Lee S.J., Kim J.H., Xuan Y.H., Lee K.H., Park S.K., Choi S.H., Chung D.I., Kong H.H., Ock M.S. (2007). Acanthamoeba: Keratopathogenicity of isolates from domestic tap water in Korea. Exp. Parasitol..

[39] Radford C.F., Minassian D.C., Dart J.K. (2002). Acanthamoeba keratitis in England and Wales: Incidence, outcome, and risk factors. Br. J. Ophthalmol..

